# A Needed Innovation for Professional Development Within Cardiovascular Fellowships

**DOI:** 10.1016/j.jacadv.2024.101557

**Published:** 2025-01-13

**Authors:** Joseph F. Nowatzke, Amit Jhaveri, Andrea M. Elliott, Julie B. Damp, Barinder S. Hansra

**Affiliations:** a Department of Medicine, Division of Cardiovascular Medicine, Vanderbilt University Medical Center, Nashville, Tennessee, USA; b Section of Critical Care Cardiology, Cardiovascular Division, University of Minnesota, Minneapolis, Minnesota, USA

**Keywords:** ACC fellows-in-training, leadership, personal growth, professional development, professionalism

**Figure:**
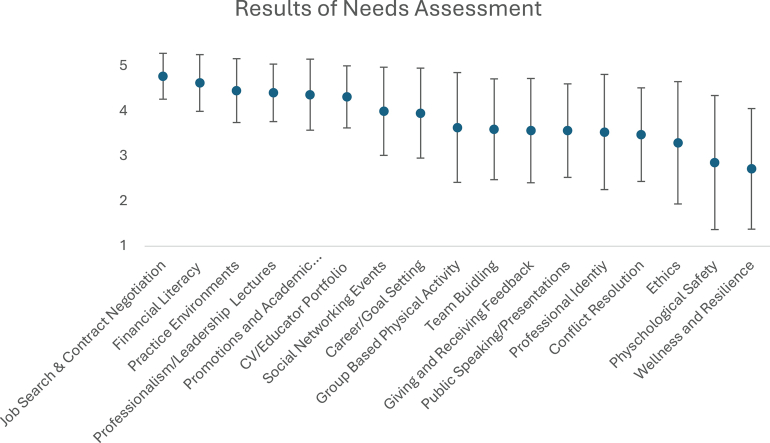

**What is the clinical question being addressed?**

How can professional development be structured and delivered while allowing for specific training programs unique interests of their trainees?

**What is the main finding?**

A needs assessment can be delivered to trainees and allow program leadership to craft a professional development curriculum to meet the specific needs identified on the needs assessment.

The primary objective of cardiology fellowship training is to provide a comprehensive academic experience designed to develop trainees into highly skilled and proficient cardiologists. The Accreditation Council for Graduate Medical Education (ACGME) has established a framework to guide all training programs toward accomplishing this goal.^1^ Additionally, the ACGME Program Requirements for Graduate Medical Education in Cardiovascular Disease clearly state the importance of professionalism.^2^ However, in contrast to other core competencies such as procedural and imaging skills, there are limited details and definitions regarding the content and implementation of a professional development curriculum. Consequently, training programs must develop their own approach to teaching nonclinical skills that are important to successful practice. Without a uniform structure, graduating fellows have a variable level of preparedness to enter independent practice.

## Methods

To assess current preparedness in professional development and optimize fellow engagement with future curriculum, we developed a 27-question needs assessment distributed among general cardiology fellows at a single training center. No ethical approval was needed given anonymous responses from participants. Similar models have been proposed for faculty development among physician educators^3^ and chief cardiology fellows.^4^

## Results

Questions were aimed at fellow self-assessment of preparedness in professional and leadership skills and fellow interest in predefined professional development topics. Twenty-eight of the thirty-seven eligible fellows completed the survey, including 8 of 9 incoming fellows, 6 of 9 first-year fellows, 8 of 9 second-year fellows, and 6 of 10 third-year fellows.

## Analysis

**Figure 1:**
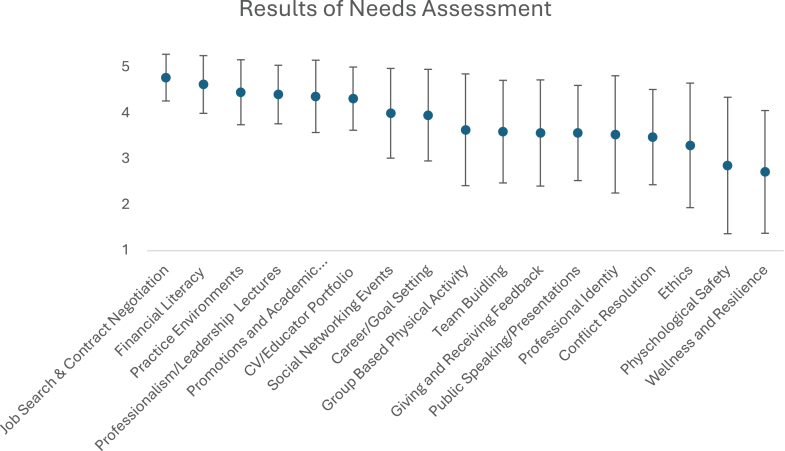
**Results of Fellowship Wide Needs Assessment to Determine Structure of Professional Development Lectures** (5 = very interested in attending a lecture on topic; 1 = least interested in attending a lecture on topic; error bars represent standard deviation).

Eleven out of twenty-eight (40.3%) fellows reported they did not feel current fellowship training had adequately provided them skills to be a leader in the field of cardiology. There was interest in a broad range of topics, as illustrated in Figure 1. In response to open-ended questions, fellows expressed desire for diversity in speakers, including those from outside of academic medicine to bring various perspectives into their training.

These findings suggest among cardiovascular fellows in our program a need for enhanced professional development opportunities. Given the lack of an established framework for professional development curricula in cardiovascular fellowship, we propose a 3-year rotating curriculum focusing on topics felt to be of most interest to fellows. This curriculum schedule will ensure all graduating fellows are exposed to key aspects of professional development, including ethics, wellness and resilience, along with contract negotiations, practice environments, financial literacy, professional achievement, and promotions.

## Conclusions

Our evaluation of professionalism and leadership readiness among trainees to our knowledge is the first of its kind. Cardiology fellowships provide robust didactic education via structured and standardized curriculum based on ACGME requirements. However, the same is not true for the topic of professionalism and leadership.

Our manuscript has several strengths. First, to address the barriers posed by the significant clinical and personal demands on fellows’ time, we developed a variety of learning opportunities in the targeted topics, with sessions built into and outside the standard fellowship didactic schedule. For example, activities within the standing schedule include traditional didactic series and less traditional formats such as “fireside with faculty.” Activities outside of the workplace include career nights, dedicated social activities with both fellows and faculty, and group exercise sessions. By incorporating diverse settings beyond the hospital environment, we aim to offer fellows valuable networking opportunities that may not otherwise be accessible and more flexible and active, hands-on learning experiences. Second, this curriculum represents and addresses the exact skills our trainees are seeking. To ensure our curriculum is dynamic and remains valuable, postsession evaluations are completed. Finally, we recognize cardiology fellowship training creates well-rounded clinicians, we believe our curriculum will help them contribute positively to health care systems and a broader community.

Limitations of our manuscript include a 75% response rate to the survey among trainees. Additionally, our small sample size at a single institution that may not represent the needs’ of trainees across the nation. However, this initiative represents a unique and novel effort to develop a professional development curriculum based directly on fellowship feedback, aiming to more comprehensively prepare fellows for independent practice, navigate team dynamics, and foster innovation. We anticipate that this framework can be adapted and applied broadly to different fellowship programs based on their unique needs. Future directions of this work would include multicenter assessments to determine what a broader range of trainees would determine to be valuable professionalism and leadership topics needed in their training.

## Funding support and author disclosures

The authors have reported that they have no relationships relevant to the contents of this paper to disclose.
